# Process combination for mechanical co-processing of residues from municipal solid waste treatment

**DOI:** 10.1177/0734242X251352808

**Published:** 2025-07-28

**Authors:** Paul Demschar, Thomas Kremlicka, Severin Rettinger, Roland Pomberger, Klaus Philipp Sedlazeck

**Affiliations:** Chair of Waste Processing Technology and Waste Management, Montanuniversitaet Leoben, Leoben, Austria

**Keywords:** MSW treatment, waste incineration, mechanical-biological treatment, mechanical waste treatment, MSWI slag processing, sensor-based sorting

## Abstract

For managing municipal solid waste (MSW), there are two different treatment methods established: thermal recovery in waste incineration (MSWI) and mechanical-biological treatment (MBT). Both generate valuable fractions and treatment residues. In the case of valuable fractions, high-calorific material from MBT is used in MSWI. Therefore, the methods are connected at the *level of waste processing*. However, there are differences in handling the treatment residues. Although the processing of MSWI slags for the recovery of metals and, to some extent, glass is already an established practice in countries like Austria, Germany, Switzerland, the Netherlands and Denmark, only a portion of these residues is landfilled. In contrast, MBT rotting residues are exclusively landfilled to date. This work addresses the development of a mechanical pre-treatment process aimed at separating a partial stream from MBT rotting residues. The resulting material was subsequently evaluated for its suitability for treatment using established MSWI slag processing technologies. The experiments demonstrated that this partial stream can be integrated into existing MSWI slag processing systems, enabling the recovery of metals (1 wt-%), glass (7.9 wt-%) and ceramics/stone (4.3 wt-%). In addition, a high-calorific refuse-derived fuel-feedstock comprising 5 wt-% was generated. The largest portion, accounting for 65.3 wt-%, consists of organic material that is currently being investigated as a potential feedstock for pyrolysis applications. The results show that appropriate pre-treatment can reintegrate 28.4 wt-% of the MBT rotting residues to the material cycle using existing infrastructure. This represents a connection between MSWI and MBT at *the level of residue treatment* for the first time.

## Introduction

In 2004, Austria implemented a regulatory ban on the landfilling of untreated municipal solid waste (MSW; Bundesministerium für Land- und Forstwirtschaft, Umwelt und Wasserwirtschaft, 2008). As a result, two distinct treatment processes have developed in parallel: thermal treatment in municipal solid waste incineration plants (MSWI plants) and mechanical-biological treatment (MBT) in specialized MBT facilities. MSW as mentioned comprises a heterogenous mixture of waste not subject to separate collection. It originates from private households, schools, administrative institutions and commercial and industrial enterprises. Excluded from MSW are separately collected recyclables, organic waste (food waste), bulky waste, end-of-life electrical and electronical equipment, spent batteries, hazardous wastes and street sweepings (Bundesministerium für Klimaschutz, Umwelt, Energie, Mobilität, Innovation und Technologie, 2023). Both treatment methods contribute to substantial reductions in waste volume, stabilize of hazardous components and produce residues suitable for safe landfill disposal, such as incineration ashes, slags and treated landfill material (Eggenberger et al., 2004; Kranert, 2017). With a share of 86 wt-% (54 wt-% directly without mechanical pre-treatment) in 2020, thermal treatment represents the dominant method for managing MSW in Austria. Currently, in Austria, 11 MSWI plants are operated, divided into 7 plants using grate incineration technology and 4 plants operating with fluidized bed incineration technology. With a treatment volume of 216,000 tonnes, a smaller amount of approximately 10 wt-% of Austria’s MSW was treated in 14 MBT plants in 2020 (Bundesministerium für Klimaschutz, Umwelt, Energie, Mobilität, Innovation und Technologie, 2023).

This bifurcation of MSW treatment is evident in most European countries. In a broader European context, it becomes apparent that Central and Western European nations with highly developed waste management systems rely predominantly on MSWI, whereas in Southern and Eastern European countries, MBT is widespread. For example, the Netherlands having a direct incineration share (without pre-treatment) of 42 wt-%, Denmark of 53.9 wt-% and Germany of 30 wt-%, making them comparable to Austria. In addition, all these countries running separate collection for organic waste/food waste (European Environment Agency, 2025). Even in countries with highly developed waste management systems – such as Austria and Germany – MBT still makes a significant contribution to MSW processing, accounting for approximately 10 wt-% (European Environment Agency, 2025). In light of the imperative for resource-efficient waste management, it is necessary to optimize and enhance these MBT processes. This study adopts a novel, system-oriented approach to link the MSWI and MBT processes, using Austria’s MSW treatment framework as a foundation. The focus lies at the *level of treatment residues*, since both processes generate residues that have, until now, been partially or entirely disposed of in landfills.

For residues out of MSWI, the terms slag and ash are both commonly used in literature. A clear distinction cannot be made due to temperature range in MSWI and the heterogenous characteristics of the MSWI residues. In the context of this study, the term slag will be used consistently in accordance with the Austrian Waste Catalogue Ordinance (Bundesministerin für Klimaschutz, Umwelt, Energie, Mobilität, Innovation und Technologie, 2020). In grate incineration, slag discharge typically occurs in a wet extractor. The rapid cooling of the residues facilitates the formation of mineral phases characterized by a substantial proportion of glassy phases (Simon and Holm, 2013). These MSWI slags are also referred to as grate ash (Simon and Scholz, 2024). In grate incineration, approximately 20–25 wt-% of MSWI slag is produced relative to the amount of treated waste (Mühl et al., 2023). In fluidized bed furnaces, combustion residues (MSWI bed ash) pass through the nozzle plate and are discharged in a dry state from the lower part of the cylindrical furnace (Bilitewski, 2008). The slag yield in this process is slightly lower compared to grate incineration, typically amounting to approximately 10 wt-% (Mühl et al., 2023). Due to lower combustion temperatures and the dry discharge, surface encrustations are not typically observed in MSWI bed slag, yielding a positive effect with respect to the recovery of metallic and mineral resources (Thomé-Kozmiensky, 2013).

In the parallel MBT process, a combination of mechanical and biological treatment is employed (Skuntan and Brunner, 2005). In the mechanical process stage, metals and various high-calorific fractions are separated (Kranert, 2017). The biogenic residue from this mechanical processing step is biologically stabilized in a rotting phase of up to 12 weeks, ensuring that the final rotting residue (MBT landfill fraction, MBT rotting residues) can be technically safely and legally deposited in mass waste landfills (Neubauer and Öhlinger, 2006).

In 2020, 2.5 million tonnes of waste were treated in Austria’s 11 MSWI plants, resulting in 513,800 tonnes of secondary waste in the form of MSWI slags, the 14 MBT plants generated about 47,000 tonnes of rotting residues (Bundesministerium für Klimaschutz, Umwelt, Energie, Mobilität, Innovation und Technologie, 2023). The management of these residues differs markedly, not only in Austria but also across Europe. The processing of MSWI slags is a well-established practice in countries such as Austria, Germany, Switzerland, the Netherlands and Denmark. In Austria, the emphasis lies on the recovery of metals and, to a lesser extent, glass; the residual mineral fraction which is about 90% of the MSWI slag must be landfilled, as its use in construction materials is prohibited by current regulations (Mühl et al., 2023). In contrast, legislation in other countries as Denmark or the Netherlands permits the utilization of the mineral fraction as road construction material or as manufactured aggregate in concrete (Alam et al., 2017; Astrup, 2007). As of 2020, six stationary MSWI slag processing facilities are operated in Austria for demetallization and to some extent glass recovery out of MSWI slags (Bundesministerium für Klimaschutz, Umwelt, Energie, Mobilität, Innovation und Technologie, 2023).

Rotting residues out of MBT are, following the biological stage, exclusively disposed of in landfill (Bundesministerium für Klimaschutz, Umwelt, Energie, Mobilität, Innovation und Technologie, 2023; Wartha et al., 2020). No standardized or widely established methods currently exist for the treatment of MBT rotting residues, neither in Austria nor internationally. Ongoing research is exploring pyrolysis as a potential alternative to conventional biological treatment (Graça et al., 2024; Wartha et al., 2020). However, there are currently no viable applications for reintegrating the rotting residue into the resource cycle.

This work presents the development of a mechanical pre-treatment process for MBT rotting residues, aiming to isolate a partial stream suitable for further processing in existing MSWI slag treatment plants. To evaluate the suitability of the developed pre-treatment process, the resulting fractions were subjected to treatment steps commonly applied in MSWI slag processing facilities (Mühl et al., 2023). Demetallization was carried out using magnetic separation (both low- and strong-field) as well as eddy current separation. The separation of mineral components (ceramics/stone) from glass was performed using sensor-based sorting. Additionally, a density separation unit was used to establish a density cut-off, allowing for the removal of light materials. The aim of these investigations is twofold: firstly, to quantify the recoverable resource potential and to characterize the material from a mechanical processing perspective; secondly, to design a pre-treatment process that enables the targeted recovery of valuable fractions. The subsequent demetallization and glass separation using standard MSWI slag treatment methods serve to validate the performance and practical feasibility of the developed approach.

**Figure 1.**
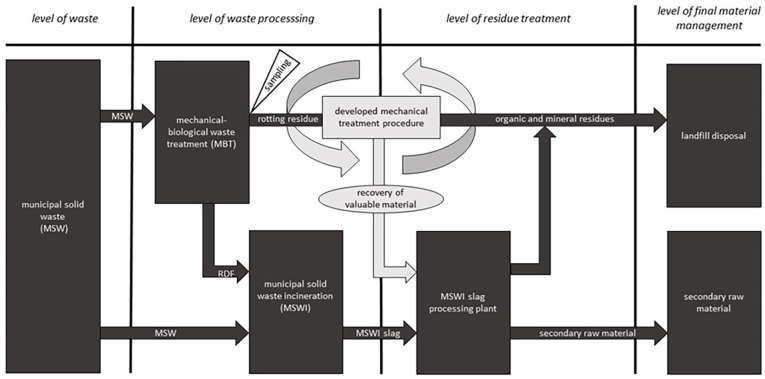
Scheme of the treatment processes for MSW in Austria including a waste management-related classification of the linkage between MBT and MSWI at the level of residue treatment, achieved through the mechanical process step developed. The sampling location is indicated. MSW: municipal solid waste; MSWI: municipal solid waste incineration; MBT: mechanical-biological treatment.

In MBT plants, recoverable resources are extracted from the waste stream during the mechanical processing stage (Kranert, 2017). This results in fractions of plastics (i.e. PET bottles) and metals for recycling, as well as high-calorific fractions for producing refuse-derived fuels (RDFs; Prochaska et al., 2004). These high-calorific fractions are thermally utilized in cement plants or MSWI plants (Schmidt, 2020). This results in a technical and systematically waste management-related linkage of MSW treatment in MSWI and MBT at the *level of waste processing* (Figure 1).

The approach presented in this work enables, for the first time, a systematic integration of the two established MSW treatment methods MBT and MSWI at the *level of residue treatment*. This integration is made possible through, on the one hand, the development of a mechanical pre-treatment process for MBT rotting residues, and on the other hand, the evaluation of its functionality and performance using existing MSWI slag treatment technologies, including demetallization and the separation of glass and ceramic/stone fractions. This represents a holistic approach and constitutes a novel contribution to waste management science, as illustrated in Figure 1.

## Material and methods

### Origin of samples

The experiments were conducted using the MBT rotting residues from an Austrian MBT plant. This facility is authorized to process 15 different waste fractions classified by waste codes in accordance with the Austrian Waste Catalogue Ordinance (Bundesministerin für Klimaschutz, Umwelt, Energie, Mobilität, Innovation und Technologie, 2020). Of the total input masses, 36% is allocated to waste code 91307 *fractions prepared for biological treatment and disposal*, 15.5% to waste code 91101 *municipal waste and similar commercial waste*, 14.4% to waste code 91103 *residues from mechanical waste processing* and the remaining capacity consists of sewage sludges (waste codes 92212 and 94501). The input is organized in three separate lines. Mechanically pre-treated fractions are already screened at 80 mm, consequently, the input material falls within the grain size class 0–80 mm (Neubauer and Öhlinger, 2006).

The MBT-process in this plant starts with screening at 35 mm. The oversized fraction (>35 mm) is rich in high calorific material for further processing in various quality grades in other facilities. Material in the grain size class of 0–35 mm is biologically treated in a three-step process (rotting stages I to III). In rotting stages I and II, rotting tunnels with dimensions of 6000 × 6000 × 26,000 mm (*w* × *h* × *l*) are filled to a maximum height of 2500 mm, enabling intensive rotting over a period of 12–13 days. The rotting process is automatically controlled, based on oxygen content, temperature, air recirculation and air supply, as well as material moisture. The temperature during the rotting process initially peaks at approximately 60°C. The course of the process, an average temperature of 47°C is maintained. The tunnels are pressure-aerated through concrete split floors, with air extraction at the ceiling and are moistened using injection nozzles. The rotting stages I and II do not differ in terms of process technology. The division serves the purpose of transferring the material between two tunnels, thereby ensuring thorough mixing. After the first rotting stage, the material exhibits a moisture content of 25 wt-%. In the second rotting stage, the moisture content decreases to a range of 15 wt-% to 20 wt-% (Neubauer and Öhlinger, 2006).

Following this stage, the material is transferred via loader to the final rotting stage, which occurs on intensively aerated composting pads, where the moisture level is adjusted to 25 wt-%. After completion of the final rotting, the material gets landfilled directly at the facility site (Neubauer and Öhlinger, 2006).

### Sampling

For processing investigations at laboratory and pilot-plant scale, material designated for landfilling and generated over 7 working days was sampled four times between May 2023 and January 2024. Figure 1 shows the specific point within the MSW management process where sampling was conducted. During this period, small quantities (approximately 4 kg each) were extracted twice for laboratory investigations, and larger quantities (approximately 500 kg each) were extracted twice for pilot-plant scale trials.

The sampling of laboratory samples was conducted in accordance with Austrian Standard S 2127, around the perimeter of the pile. The depth of the sampling probes was varied as specified by the Standard. Prior to sampling, the top 300 mm of material along the edges was scraped off using an uncoated stainless-steel shovel. Additionally, several compartments were extracted from the middle of the heap using an excavator bucket to ensure a representative cross-section of the pile. A total of 10 subsamples were collected. The mass of the subsamples was calculated in accordance with Austrian Standard S 2127 based on the dimensions of the biggest grain in the heap (Austrian Standards Institute, 2011). The subsamples were then reduced according to the standard procedure of quartering and combining to create a qualified subsample (Länderarbeitsgemeinschaft Abfall, 2001). In this manner, three parallel samples were obtained: two for laboratory investigations and one as a reserve sample.

For the pilot-plant scale trials, where approximately 500 kg material was required, sampling was conducted mechanically using an excavator due to the large quantities needed. As with the laboratory samples, great care was taken to adhere strictly to Austrian Standard S 2127. In this case, 10 subsamples were combined to form the final qualified subsample (Austrian Standards Institute, 2011).

### Laboratory treatment investigations

In the laboratory experiments, sieve analyses were conducted using a *Retsch AS 400 Control* sieve shaker. This process produced grain size classes of 0–5, 5–8, 8–14 and 14–35 mm. These fractions were subsequently classified into categories including *glass, ceramics/stone, plastics* and *others* through hand sorting. This procedure was carried out twice: once with fresh, process-moist material and once with material that had been dried to constant weight prior to the investigations. The drying process was carried out in a drying oven at 105°C until constant weight was achieved, allowed for the determination of the moisture content. This procedure was executed and documented in accordance to Austrian Standard EN 14346 (Austrian Standards Institute, 2007).

By comparing the process moist and dry samples, the influence of material moisture content on the distribution of valuable materials across grain size classes could be assessed. These findings provide key insights for designing an optimized processing method. In the following sections, unless explicitly stated otherwise, valuable materials, refer to *glass, ceramics/stone* and *plastics*. Metallic components were present only in small amounts and were therefore assigned to the sorting fraction labelled *others*. Measurements were obtained by weighing the generated fractions and calculating mass balances based on the collected data.

Subsequently, the recovery rate *r_ij_* was calculated according to equation (1; Schubert, 1988):

(1) $$r_{ij}=\frac{r_{mj}*g_{ij}}{g_{i0}}*100\;[\%]$$

where *r_ij_* is the recovery rate of component *i* in product *j, r_mj_* is the mass yield of product *j, g_ij_* assay of component *i* in product *j* and *g*_0_ is the assay of component *i* in feed.

The assay of component *i* in product *j* refers to the proportion of a specific component within a defined property class (e.g. grain size class). For instance, it describes the share of *glass* contained in the grain size class 5–8 mm. The assay of component *i* in the feed indicates the proportion of that component in the total input material. For example, it reflects the overall share of *glass* in the MBT rotting residues. Accordingly, the recovery rate specifies the fraction of a component (e.g. *glass*) from the feed that is transferred into a defined product class (e.g. grain size class 5–8 mm).

### Pilot-plant scale investigations: Pre-treatment and demetallization

Based on the results of the laboratory investigations, experimental plans were developed and trials conducted at pilot-plant scale at IFE Aufbereitungstechnik GmbH between 29 January and 2 February 2024. The processing units applied in the experiments were all manufactured by IFE Aufbereitungstechnik GmbH. The technical specifications of the machines used in the pilot-plant scale investigations are provided in Table 1 (IFE Aufbereitungstechnik GmbH, 2024).

**Table 1.** Technical specifications of the mechanical processing machines used for pilot-plant scale investigations.

Process stage	Machine	Parameters	Values
Pre-screening	Flip-flop screen	Labelling	IFE Trisomat
		Dimensions sieve surface (width × length)	800 × 3600 mm
		Cut point	5 mm
		Inclination	18°–24°
		Acceleration	Up to 30 × g m s^−2^
		Screen lining	Polyurethane sieving mats
Washing screening	Dewatering screen	Labelling	IFE dewatering screen
		Dimensions sieve surface (width × length)	400 × 1000 mm
		Cut point	5 × 15 mm (rectangular meshes)
		Amplitude	5–10 mm
		Inclination	3° upwards
		Overflow weir	20 mm
		Screen lining	Polyurethane system panels
Density separation	Air separating table	Labelling	IFE-Sort
		Dimensions (width × length)	1000 × 1000 mm
		Inclination	4°–20°
Magnetic separation – weak field	Weak field drum separator	Labelling	IFE high speed drum separator type HPG
		Drum diameter	500 mm
		Drum width	650 mm
		Magnetic flux density	100–220 mT
		Magnetic technology	Barium ferrite magnet
Eddy current separation	Eddy current separator	Labelling	IFE eddy current separator type STRATOS
		Belt width	500 mm
		Belt speed	up to 2.5 m s^−1^
		Technology	eccentric eddy current separator
		Pole changing frequency	180–750 Hz
		Magnetic flux density	300–370 mT
Magnetic separation – strong field	Strong field drum separator	Labelling	IFE high intensity drum separator type KHP
		Drum diameter	500 mm
		Drum width	650 mm
		Magnetic flux density	850–1000 mT
		Magnetic technology	Neodymium magnet

### Sensor-based sorting investigations

The *glass/ceramics/stone*-fraction (GCS-fraction) generated during the laboratory investigations was further sorted into *glass* components and *ceramics/stone* components using sensor-based sorting technology. Prior to this step, the GCS-fraction underwent a fundamental process engineering characterization through sieving and manual sorting. Using a Retsch AS 400 Control sieve shaker, the material was divided into grain size classes 0–5, 5–8, 8–14 and 14–35 mm. Subsequently, the material was hand sorted to create the material groups *glass, ceramics/stone, plastics* and *others* (i.e. wood, metals). The sorting trials were conducted on the sensor-based sorting station at the Chair of Waste Processing Technology and Waste Management at University of Leoben, Austria. The sorting station, type CLA5 2w/R-T/VIS-NIRH/S/6,2/B, was manufactured by Binder+Co AG (2017). It is designed for a grain size ranging from 5 to 20 mm and from 20 to 100 mm with a feed capacity of 0.1–0.3 tonnes hour^-1^. The pneumatic nozzles used for material ejection operate at an adjustable air pressure of 2–9.5 bar, which is controlled via an external barometer (Binder+Co AG, 2017).

The sorting station is equipped with a AViiVA^®^ SC2 CL Camera Link^®^ colour linescan camera manufactured by Teledyne e2v. This camera includes a charge-coupled device that can also be used to create sorting recipes for colour separation (Teledyne e2v, 2009). The sensor unit is equipped with a hyperspectral camera from EVK DI Kerschhaggl GmbH (2017).

The trials were conducted using the transmission method, in which the sensors are configured so light-transmitting particles (*glass*) are not ejected. All other particles are detected and pneumatically ejected (*ceramics/stone*; Faist and Ragossnig, 2008). The air pressure was adjusted according to the grain size class, ranging from 5 bar for the 5–8 mm fraction to 9 bar for the 14–35 mm fraction. This adjustment ensured that even heavy and large particles are ejected with sufficient force to achieve a precise separation. The resulting particle classes were categorized as *Pass* (*glass*) and *Eject* (*ceramics/stone*).

The sensor-based sorting trials were evaluated by determining mass balances and manually sorting the output into the categories *correct* and *incorrect*. Incorrect particles were then weighed to quantify sorting accuracy.

## Results and discussion

### Distribution of valuable materials in grain size classes

The process moisture of the MBT landfill fraction was determined at 25 wt-%. Processing proceeded without any complications, with no occurrences of sieve mesh clogging or issues related to trapped particles. To calculate the recovery rates (equation (1)), all material within the grain size classes 14–35 and 8–14 mm was manually sorted. For the grain size class 5–8 mm, a sample was taken after one reduction step by quartering and combining, as specified by Länderarbeitsgemeinschaft Abfall (2001). In the 0–5 mm grain size class, this reduction process was repeated three times. Consequently, the size of the sorting sample represented 1/8 of the original sample material.

**Figure 2.**
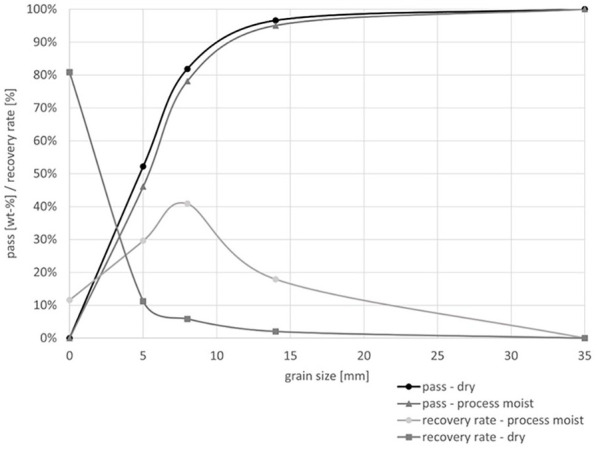
Synopsis of grain size specific recovery rates compared for the dry and process moist conditions.

In the process moist state, compared to the dry material, there is a slight shift in the grain size distribution towards coarser grain sizes. This shift is attributed to adhesion of fine grains to the surfaces of larger grains due to the process moisture. As a result, fine grains are not exposed and cannot be separated by sieving. The shift in valuable material phases towards coarser grain sizes is much more pronounced than the change in the overall grain size distribution. The mass yield in the cumulative grain size class 5–35 mm is 2 wt-% higher in the process moist condition compared to the dry condition. Simultaneously, the recovery rate for the valuable material phases is 23% higher in the process moist state. This shift in material yield, as a function of the grain size distribution, is visualized in Figure 2.

**Figure 3.**
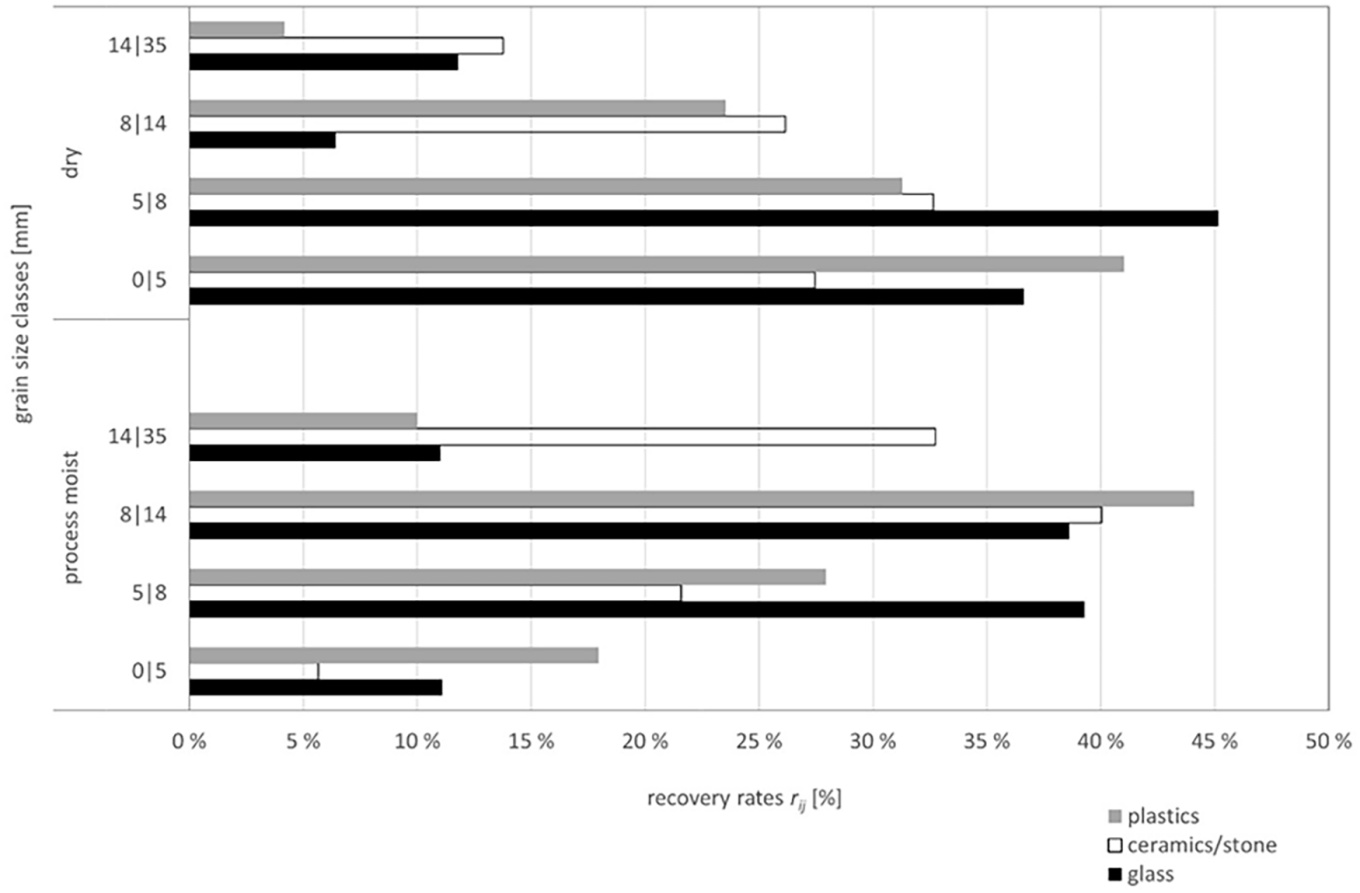
Changes in recovery rates as a function of process moisture for components defined as valuable materials. Plotted for each of the investigated grain size classes.

The change in recovery rates based on grain size distribution due to the material’s process moisture leads to a separation effect between valuable materials and undesired accompanying phases. A significant 88% of the valuable materials are located in the coarse components (>5 mm), whereas the accompanying phases are primarily found in the finest fraction <5 mm. In terms of mass, the finest fraction dominates, comprising 81 wt-%. Despite the much smaller mass of 19 wt-%, a considerable concentration of valuable material is present in the cumulative grain size class 5–35 mm. The results of the sorting analyses, which highlight the shift in the distribution of valuable materials between the dry and process-moist conditions, are shown in Figure 3.

The mass yield of the grain size class 14–35 mm is 5 wt-%. In the process moist condition, the recovery rate of *ceramics/stone* increases by 19%, reaching 32.7%. Additionally, 11% of the *glass* content is transferred to the grain size class 14–35 mm. The recovery rate of *plastics* in the process moist condition increases by 6%, amounting to 10% in this grain size class. In conjunction with the low mass yield values, the recovery rates for *glass, ceramics/stone* and *plastics* suggest that the fraction 14–35 mm serves as an enrichment fraction for these valuable materials. This can be attributed to the differing surface characteristics of *glass* and *ceramics/stone*. Although *glass* has a smooth surface, *ceramics/stone* materials also contain clay shards with rough surfaces, which promote the formation of agglomerates or the attachment of fine, moist particles. The increase in recovery rates of *ceramics/stone* by 19% and the near-constancy recovery rate of *glass* (decrease by 0.8%) support this explanation. For *plastics*, the increase in recovery rate is attributed to the flat shape of the film particles, which also tend to form attachments and nests.

For the mass yield of the grain size class 8–14 mm, no significant differences are observed between the dry and process moist conditions. In both cases, the mass yield is approximately 11 wt-%. However, the recovery rates change, with the fraction 8–14 mm containing 38.6% of *glass* and 40% of *ceramics/stone*, which accounts for the majority of the mineral valuable materials. The recovery rate of *plastics* in this grain size class is 44%, representing an increase of 21% compared to the dry condition.

In grain size class 5–8 mm, the mass yield slightly increases compared to the dry material condition, reaching 15.4 wt-%. Despite the low mass yield, the recovery rates of *glass* (39.3%), *ceramics/stone* (21.6%) and *plastics* (28%) remain high. However, this grain size class is the only one where the recovery rates for *glass, ceramics/stone* and *plastics* are lower in the process moist condition compared to the dry condition. This observation demonstrates the impact of moisture content, which induces a shift in the recovery rates of these valuable materials towards coarser grain sizes.

In the finest grain size class considered, 0–5 mm, there is a negligible reduction in mass yield by 2.4 wt-%, resulting in 68 wt-% in the process moist condition. This fraction, therefore, represents the largest mass fraction in both, the process moist and dry conditions. However, the recovery rates for valuable phases – *glass* (11.1%), *ceramics/stone* (5.7%) and *plastics* (18%) – are lower in this fraction due to the shift of valuable materials towards coarser grain size classes.

For scaling the experiments to the pilot-plant scale for recovering valuable materials, the following conclusions can be drawn:

- Wet processing is essential: To exploit the advantageous distribution of valuable materials observed in the moist condition, the material must be processed under wet conditions.
- Pre-separation of fine fractions: The 0–5 mm contains a low proportion of target fractions and should be pre-separated using screening techniques to enhance process efficiency.
- *Plastics* recovery through density sorting: Plastics are not easily separable from *glass* and *ceramics/stone* by sieving and must be recovered through density sorting. Moreover, the separation of *plastics* is not part of the process in existing MSWI slag processing plants, which is why this step must be integrated into the MBT rotting residues pre-treatment process.

### Mechanical treatment experiments: Pre-treatment and demetallization

**Figure 4.**
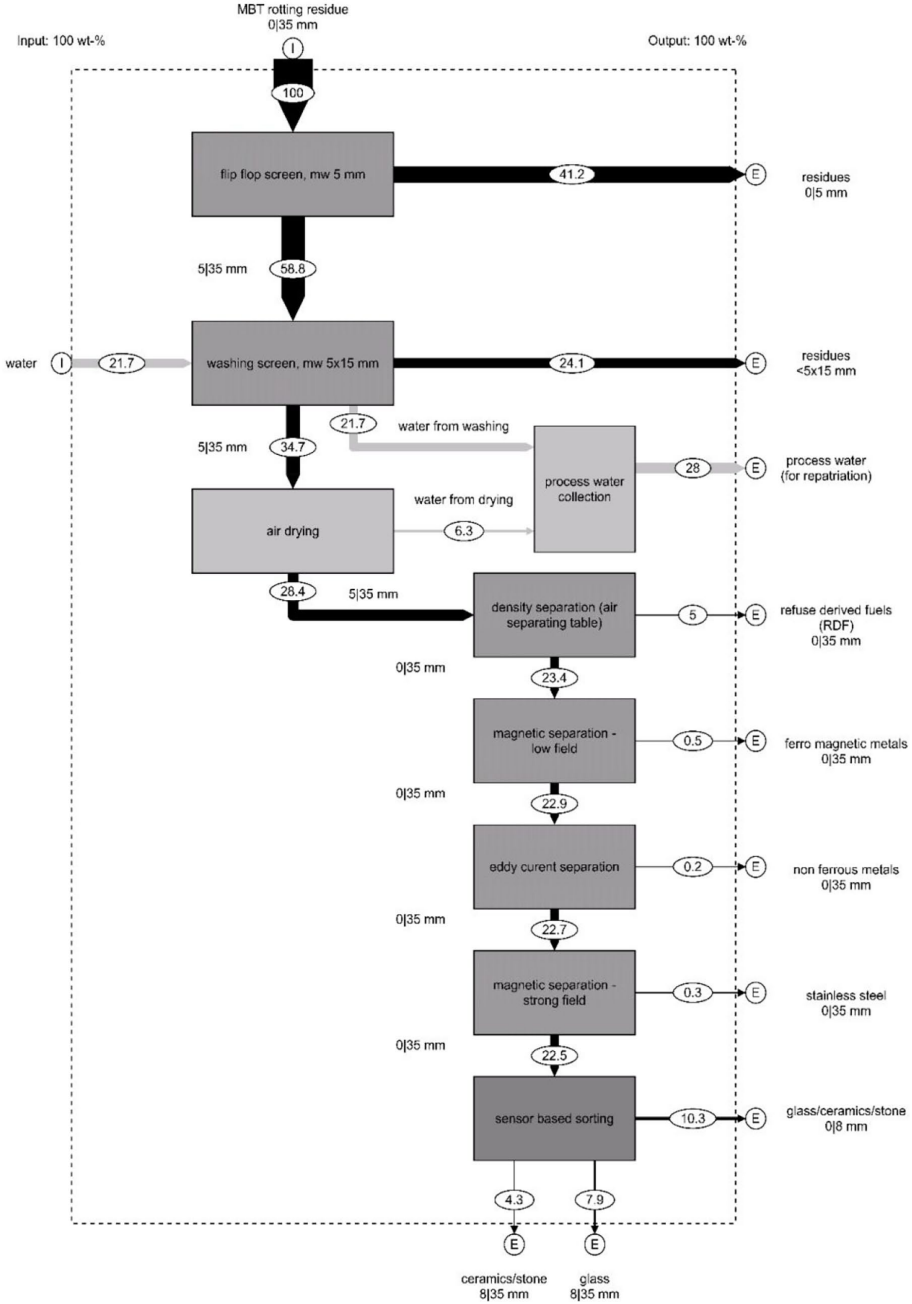
Flow chart of the developed mechanical process for pre-treatment and recovering valuable materials from MBT rotting residue. Input streams (I), output streams (E) and all other streams represent mass balances in wt-% relative to the total input of original MBT rotting residue. MBT: mechanical-biological treatment.

These investigations pursue two objectives: firstly, a partial stream is to be separated from the MBT rotting residues and processed in such a way that it becomes suitable for input into existing MSWI slag treatment plants. This is achieved with preliminary screening at 5 mm, a washing step including dewatering and density separation using an air separating table. Secondly, the experiment is designed to directly evaluate the suitability of the recovered valuable materials for processing with established MSWI slag treatment technologies. This includes demetallization via two-stage magnetic separation, eddy current separation and, as the final process step, sensor-based sorting for glass removal. A balanced flow diagram of the pilot-plant scale process is shown in Figure 4.

The MBT rotting residues undergo an initial screening process using a flip flop screen with a 5 mm mesh size. In the retained 5–35 mm fraction, valuable materials are embedded in a matrix with strongly adhesive, earthy characteristics. The process moisture content of 25 wt-% contributes to significant agglomerations and pronounced adhesions on the surfaces of valuable particles. This compromises the precision of the initial screening at 5 mm, as fine particles adhere to valuable surfaces, obstructing efficient separation. The effectiveness of screening diminishes with finer mesh sizes and lower screen dynamics. Fine mesh sizes are particularly prone to clogging due to the abrasion of finer particles, forming a layer on the screen surface that inhibits classification. Machines with low dynamics fail to mitigate these effects effectively.

To dislodge adhered fine particles, the material is processed using a dewatering screen equipped with intense spraying mechanism across its surface. This step achieves a superficial washing effect that releases surface adhesions. The material, spread in a monolayer by a dosing bunker onto a linear vibratory screen, undergoes intense spraying to prevent mesh clogging and enable sharp separation of valuable and unwanted phases. The dewatering screen features a 20-mm height overflow weir at the discharge end, forming a triangular material bed that generates shear stress on the particles, creating an attrition effect that further cleans the particle surfaces. The underflow (wash water and sludge) is collected and combined with the 0–5 mm fraction from the initial screening step. This fraction, accounting for 65.3 wt-% of the total mass, was evaluated in accordance with the Austrian landfill ordinance for the disposal of MBT landfill fractions and was found to meet all relevant limit values, making landfilling permissible (Bundesministerium für Land- und Forstwirtschaft, Umwelt und Wasserwirtschaft, 2008). However, material recovery is also being pursued for this fraction, which is the subject of ongoing research. Specifically, pyrolysis is being investigated to produce biochar that could be used as a CO_2_-storage medium in concrete applications.

The overflow, largely free of surface adhesions, contains the valuable materials in a liberated form. After thorough air drying, the material is subjected to density-based separation on an air-separating table. This process step is necessary because the light fraction (i.e. plastics, wood, paper) cannot be treated in MSWI slag processing plants, as it would combust during the incineration process and thus does not form part of the MSWI slag. Therefore, it must be separated during the pre-treatment stage. The density sorting process also liberates grains smaller than 5 mm. The air flow disrupts plastic nests, whereas the vibratory motion of the separation table breaks up agglomerates and removes residual adhesions, freeing fine grains. As a result, all valuable fractions, despite the initial 5 mm screening, are ultimately included within the broader grain size class 0–35 mm.

The light fraction derived from density sorting, consisting primarily of high-calorific-value plastics, is classified as a feedstock for producing RDF. Subject to further analysis, this fraction has the potential to serve as a calciner substitute fuel in cement production, aligning with established guidelines for alternative fuels in Austria (Bundesministerium für Land- und Forstwirtschaft, Umwelt und Wasserwirtschaft, 2017).

Following the pre-treatment and separation of the organic components, the heavy fraction obtained from the density sorting constitutes the GCS-fraction, which also contains metallic components. Further investigations aim to demonstrate the processability of this fraction using the treatment technologies commonly applied in existing MSWI slag processing plants. The selective recovery of metallic components is achieved via magnetic separation followed by eddy current separation. By exploiting differences in magnetic susceptibility (magnetic separation) and electrical conductivity (eddy current separation), ferrous (FE) and non-ferrous metals are recovered (Kranert, 2017). The process involves two stages of magnetic separation (low- and strong-field) followed by eddy current separation. After the removal of metallic components, the residual material comprises the GCS-fraction.

During the pilot-plant scale investigations, the following fractions were generated:

- Residual fraction 0–5 mm: Fine material remaining after initial screening (currently pyrolysis investigations).
- Residual fraction <5 × 15 mm (sedimented solid from wash slurry): Solid material obtained from the sedimentation of slurry water from the washing process (currently pyrolysis investigations).
- RDF fraction 0–35 mm: RDF feedstock potentially suitable for energy recovery applications.
- Strongly magnetic FE fraction 0–35 mm: Ferrous metals with high magnetic susceptibility, such as stainless-steel.
- Weakly magnetic FE fraction 0–35 mm: Iron metals with lower magnetic susceptibility, including steel.
- Non-ferrous metal fraction 0–35 mm: Non-magnetic metals such as aluminium or copper, separated via eddy current separation.
- GCS-fraction 0–35 mm: Non-metallic mineral components, such as glass, ceramics and stones.

### Sensor-based sorting of GCS fraction

The demetallized GCS-fraction in the grain size range of 0–35 mm was screened and subjected to manual sorting analysis to assess its composition. The results of the recovered material distribution within this fraction are presented in Table 2.

**Table 2.** Results of screening and manual sorting of the GCS-fraction (mass balance and recovery rates according to equation (1)).

Grain size range (mm)	Mass content glass (wt-%)	Recovery rate glass (%)	Mass content ceramics/stone (wt-%)	Recovery rate ceramics/stone (%)
14–35	43.5	13.7	50.5	22.8
8–14	66	43.3	30	28.3
5–8	56.3	32.7	37.3	31
0–5	44	10.3	53	17.9

GCS: glass/ceramics/stone.

Lederer et al. (2024) discussed the removal of glass from fluidized bed incineration slags and presented a material-based and process-oriented characterization of the material. They identified the primary components (98 wt-%) are defined as mineral constituents (ceramics, stones) and glass (white glass, green glass, brown glass). Their study highlights that these components exist as free constituents in the combustion residue due to the combustion conditions within the incineration process (Lederer et al., 2024). The GCS-fraction analysed in the present study exhibits similarities to slags from fluidized bed incineration systems, both in component composition (95 wt-% mineral components) and in the physical modes of occurrence. This resemblance can be attributed, firstly, to the comparable nature of input streams in MBT plants and MSWI plants, which predominantly consist of MSW (Bundesministerium für Klimaschutz, Umwelt, Energie, Mobilität, Innovation und Technologie, 2023). Secondly, glass, ceramics and stones remain largely unaffected by the thermal combustion process in MSWI or the biogenic decomposition occurring in MBT plants (Eckardt and Lübben, 2024; Kranert, 2017). After both the treatment process implemented in this study (Figure 4) and slags from fluidized bed combustion systems, particles are free of agglomerations and surface adhesions (Kranert, 2017). In fluidized bed incineration, this phenomenon results from the dry process conditions, dry discharge and abrasive stress during combustion (Thomé-Kozmiensky, 2013). Similarly, in the present treatment process, intensive spraying on the dewatering screen effectively breaks up agglomerations and cleanses particle surfaces.

To evaluate the suitability of the produced GCS-fraction for glass separation in industrial MSWI slag processing plants, tests on sensor-based sorting were conducted. These investigations aimed to mechanically separate the GCS-fraction into distinct *glass-* and a *ceramics/stone*-fractions. The sensor-based sorting tests initially performed in the laboratory were scaled up to an industrial level, using an operational plant. This scaling step served to demonstrate the effectiveness of the treatment process outlined in Figure 4 for incorporating the MBT landfill fraction into established industrial scale processes for MSWI slag processing. By achieving this integration, a connection is established between the two major MSW treatment processes at the *level of residue treatment* (Figure 1).

The results of these sensor-based sorting trials are summarized in Table 3. The distribution of *Eject* and *Pass* is presented as a percentage of the total input stream (wt-%), whereas the purity (wt-%) refers to the composition of the respective extracted stream in *Eject* or *Pass*.

**Table 3.** Results of sensor-based sorting investigations in laboratory and industrial scale.

Grain size range (mm)	Laboratory investigations				Industrial-plant scale investigations			
	Eject (ceramic/stone)		Pass (glass)		Eject (ceramic/stone)		Pass (glass)	
	Mass fraction (wt-%)	Purity (wt-%)	Mass fraction (wt-%)	Purity (wt-%)	Mass fraction ([wt-%)	Purity (wt-%)	Mass fraction (wt-%)	Purity (wt-%)
Individual results								
14–35	57.9	99.0	42.1	94.2	35.2	95.4	8.3	57.5
8–14	24.8	98.4	75.3	82.7				
5–8	2.3	96.6	97.7	56.0	n.v.	n.v.	n.v.	n.v.
	Laboratory investigations				Industrial-plant scale investigations			
Product fraction (mm)	Mass fraction (wt-%)		Purity (wt-%)		Mass fraction (wt-%)		Purity (wt-%)	
Overall results								
8–35 glass	35.0		98.6		8.3		57.6	
8–35 ceramic/stone	19.3		86.4		35.2		95.4	
0–8 glass/ceramic/stone	45.7		n.v.		43.6		n.v.	

n.v.: no value.

In both laboratory and industrial scale trials, sensor-based sorting achieved a theoretical separation cut at 8 mm. In the laboratory experiments, the sorting purity within the 5–8 mm fraction was already low, at 56 wt-%. Furthermore, effective separation of the 0.5 mm fraction was not feasible. For the industrial scale trials, a mechanical separation at 8 mm was performed before sensor-based sorting. The findings of Mühl et al. (2023) support these results, demonstrating that sensor-based sorting of MSWI slag particles smaller than 8 mm fails to achieve sufficient quality, due to significantly reduced sorting efficiency with decreasing grain size. Although the light transmission-based sensors used in the trials can detect grains smaller than 8 mm, the coarse nozzle arrangement limits the effective ejection of these particles. Consequently, sorting for particles smaller than 8 mm was not successful in the trials.

In the *Pass* fraction, where *glass* accumulates, unejected *ceramics/ston*e particles caused contamination. Conversely, the *Eject* fraction was purer, though minor contamination occurred due to heavily soiled or coated *glass*-particles, preventing light transmission. For grains in the 8–35 mm class, 54.3 wt-% of the *glass* was present. Sensor-based sorting of this partial stream recovered 54.9% of the *ceramics/stone*-content and 54.1% of the *glass*-content from the total input.

The sorting purity for the *ceramics/stone*-fraction in the cumulative 8–35 mm grain size class was 98.6 wt-% in the laboratory trials and 95.4 wt-% for the industrial scale trials. However, the purities for the *glass*-fraction were lower, measured at 86.4 wt-% in the laboratory experiments and 57.5 wt-% in the industrial scale trials. These results highlight the challenges of achieving high sorting purity for glass during industrial scale sensor-based sorting, particularly when scaling up the process.

Sensor-based sorting within the 8–35 mm grain size class enables the production of a highly pure *ceramics/stone*-fraction and a *glass*-fraction with comparatively lower purity. However, the cumulative grain size class 0–8 mm, which cannot be effectively sorted, represents 45.7 wt-% of the total GCS-fraction by mass. This grain size class contains 45.1% of the *ceramics/stone* content and 45.9% of the *glass* content.

## Conclusion

The investigations, building upon a fundamental characterization of MBT rotting residues in terms of mechanical processing, have demonstrated the potential for resource recovery from these materials. Firstly, a pre-treatment process was developed to separate a substream containing valuable materials (glass, ceramics/stone, metals, plastics) from the organic matrix of the MBT rotting residues, representing 28.4 wt-%. In a second step, this substream was evaluated for its suitability for treatment using established processes in industrial MSWI slag processing plants. The findings confirmed that process moisture conditions shift the distribution of valuable materials towards coarser grain size classes. Screening at 5 mm concentrates 88% of the valuable materials in the 5–35 mm fraction, representing a 23% increase compared to dry conditions, which is why a wet process was chosen for pre-treatment. Following an initial screening step at 5 mm, the 5–35 mm fraction undergoes washing on an intensively rinsed dewatering screen. This allows the organic, pasty components to be separated into the underflow, whereas the valuable materials remain dissolved in the overflow material. The underflow fractions of these two processing steps, accounting for 65.3 wt-%, is depleted in valuable materials and enriched in organic components. These materials must either be deposited separately in a mass waste landfill (compliance with the Austrian landfill ordinance can be met) or directed towards further recovery. Pyrolysis trials are currently underway to enable the material to be used as biochar for CO_2_-storage in concrete.

Before the generated fraction in grain size class 5–35 mm can be tested for its suitability with existing MSWI slag processing technologies, the high-calorific, low-density fraction (composed of plastics, wood, paper) must be removed. This step is necessary because these components are combusted during MSWI and therefore do not end up in the slag, meaning the processing systems are not designed to handle them. Density-based separation on an air separation table further divides the valuable materials into a heavy glass/ceramics/stone/metals-mixed fraction and a light but high-calorific fraction for RDF production. Due to the effects on the air separation table, the grain class distribution is re-established to a range of 0–35 mm. The generated glass/ceramics/stone/metals-mixed fraction is comparable to MSWI slag in terms of both composition and macroscopic properties. Thus, the developed pre-treatment process is capable of separating a partial stream from the MBT rotting residues that can subsequently be examined for its suitability for treatment using established MSWI slag processing techniques.

The approach presented in this publication establishes, for the first time, a linkage between the established MSW treatment methods – MBT and MSWI – at the *level of residue treatment*. By selectively separating a partial stream from the MBT rotting residues, the amount of waste requiring landfilling out of MBT can be reduced by up to 28.4 wt-%. The separated residues can be treated in existing facilities and directly reintegrated into the value chain. This complies with EU legal requirements to reduce the amount of waste sent to landfill and represents an innovative approach to further developing existing MSW management systems in the context of implementing a circular economy.
